# Intracellular Delivery of Proteins via Fusion Peptides in Intact Plants

**DOI:** 10.1371/journal.pone.0154081

**Published:** 2016-04-21

**Authors:** Kiaw Kiaw Ng, Yoko Motoda, Satoru Watanabe, Ahmad Sofiman Othman, Takanori Kigawa, Yutaka Kodama, Keiji Numata

**Affiliations:** 1 Enzyme Research Team, Biomass Engineering Research Division, RIKEN Center for Sustainable Resource Science, Wako, Saitama, Japan; 2 School of Biological Sciences, Universiti Sains Malaysia, Penang, Malaysia; 3 Laboratory for Biomolecular Structure and Dynamics, RIKEN Quantitative Biology Center (QBiC), Yokohama, Kanagawa, Japan; 4 Department of Computational Intelligence and Systems Science, Interdisciplinary Graduate School of Science and Engineering, Tokyo Institute of Technology, Yokohama, Kanagawa, Japan; 5 Center for Bioscience Research and Education, Utsunomiya University, Utsunomiya, Tochigi, Japan; Consejo Superior de Investigaciones Cientificas, SPAIN

## Abstract

In current plant biotechnology, the introduction of exogenous DNA encoding desired traits is the most common approach used to modify plants. However, general plant transformation methods can cause random integration of exogenous DNA into the plant genome. To avoid these events, alternative methods, such as a direct protein delivery system, are needed to modify the plant. Although there have been reports of the delivery of proteins into cultured plant cells, there are currently no methods for the direct delivery of proteins into intact plants, owing to their hierarchical structures. Here, we demonstrate the efficient fusion-peptide-based delivery of proteins into intact *Arabidopsis thaliana*. Bovine serum albumin (BSA, 66 kDa) was selected as a model protein to optimize conditions for delivery into the cytosol. The general applicability of our method to large protein cargo was also demonstrated by the delivery of alcohol dehydrogenase (ADH, 150 kDa) into the cytosol. The compatibility of the fusion peptide system with the delivery of proteins to specific cellular organelles was also demonstrated using the fluorescent protein Citrine (27 kDa) conjugated to either a nuclear localization signal (NLS) or a peroxisomal targeting signal (PTS). In conclusion, our designed fusion peptide system can deliver proteins with a wide range of molecular weights (27 to 150 kDa) into the cells of intact *A*. *thaliana* without interfering with the organelle-targeting peptide conjugated to the protein. We expect that this efficient protein delivery system will be a powerful tool in plant biotechnology.

## Introduction

Plant genetic engineering is commonly used in plant breeding to improve the productivity and enhance crop fitness of, including yield enhancement, nutritional quality enhancement, herbicide tolerance, drought resistance, pest resistance and viral resistance [1]. Beyond crop improvement applications, plants can be engineered to exhibit high photosynthesis efficiency with the aim of enhancing carbon dioxide sequestration from the atmosphere and combating global warming [2]. Currently, plants are mainly genetically modified by the delivery of exogenous DNA encoding a desired trait via *Agrobacterium*-mediated transformation or particle bombardment. As compared to protein delivery, the DNA delivery is commonly used to modify the plant due to the ease of preparation, the stability and the smaller size of plasmid DNA. However, the DNA delivery can cause several potential problems, including the random insertion of exogenous DNA into the plant genome and the uptake of antibiotic resistance genes by pathogenic bacteria in soil via horizontal gene transfer [3]. Thus, it is crucial to modify the plant via direct protein delivery strategy. One of the technical issues related to the protein delivery is the diverse feature of different proteins such as various size, surface charge and conformation, leading to the difficulty in the design of a universal protein carrier. Moreover, proteins are more susceptible to the denaturation and loss of functionality due to their fragile tertiary structures [4, 5].

CPPs, a group of short peptides with at most 30 to 35 amino acid residues, has the ability to translocate across the cell membrane [6]. In animal system, the direct protein delivery strategy using CPPs has been extensively developed [7–10]. However, the peptide-mediated protein delivery system in plant still scarcely developed. Plant cells differ significantly from animal cells in hierarchal structure of cells. Plant cell membrane is surrounded by rigid cell wall, and plant cell wall consists of a complex network of various carbohydrates with negative charge. CPP has been found to be trapped within the cell wall due to its ionic interaction with the negatively charged cell wall components [11]. In this study, the designed CPP for plant system must be able to penetrate the cell membrane without being absorbed onto the negatively charged cell wall.

There are only few reports reporting the use of CPP for protein delivery plant cells such as corn root tip cells, tobacco BY-2 cells, microspore cultures, and onion epidermal cells [12–18]. Those reported studies were only at cellular assay level. To date, there is no report on protein delivery system in intact plants due to hierarchical structure. In this study, we attempted to develop a peptide-based protein delivery system in intact plant.

Peptide-mediated delivery strategy mainly involves the formation peptide-fusion protein, which is achieved by the formation of carrier peptide and protein of interest by chemical cross-linking or by cloning followed by expression [19]. However, the drawbacks of these strategies are labor-intensive, time-consuming, and loss of biological activity of protein cargoes [20]. As an alternative to the covalent-linking strategy, we propose a new strategy for protein delivery in plant based on electrostatic interaction between a carrier peptide and protein with the opposite charge. The advantages of this non-covalent strategy are the ease of use, versatility with respect to various protein cargoes and preservation of biological activity of protein.

**Fig 1:**
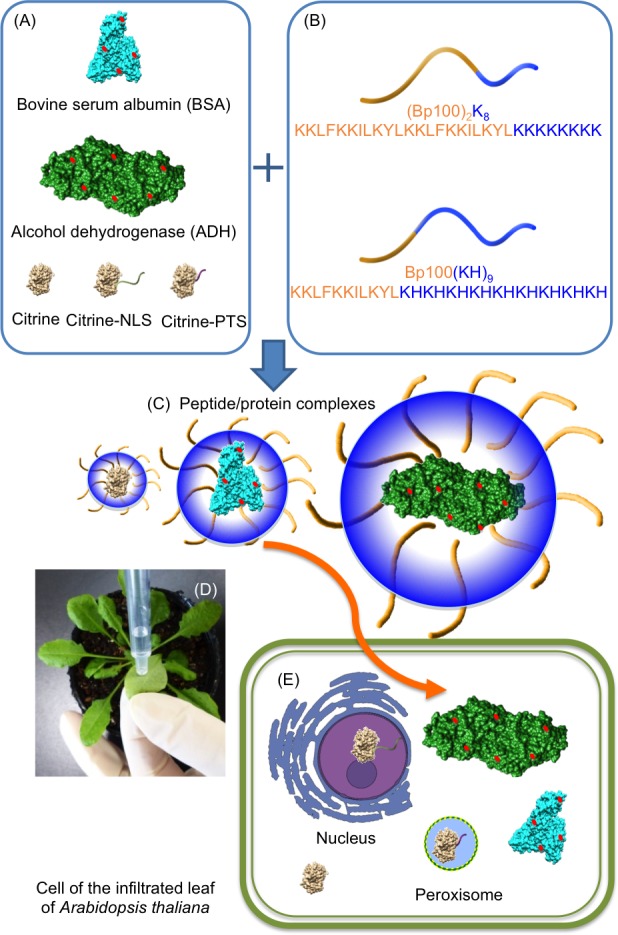
Scheme of the fusion-peptide-mediated intracellular delivery of protein cargoes into the leaves of intact *A*. *thaliana*. (A) The protein cargoes were as follows: bovine serum albumin (BSA) (66 kDa), alcohol dehydrogenase (ADH) (150 kDa), Citrine (27 kDa), Citrine with nuclear localization signal (Citrine-NLS) (27 kDa) and Citrine with peroxisomal targeting signal (27 kDa) (Citrine-PTS). BSA and ADH were labeled with Rhodamine B isothiocyanate (red circle). Citrine, Citrine-NLS and Citrine-PTS are fluorescent proteins. (B) The cell-penetrating peptide was BP100, and the polycationic peptides were K_8_ or (KH)_9_. The designed fusion peptides were (BP100)_2_K_8_ and BP100(KH)_9._ (C) Each protein was mixed with (BP100)_2_K_8_ or BP100(KH)_9_ at various peptide/protein molar ratios to form the peptide/protein complexes. (D) Infiltration of peptide/protein complexes into *A*. *thaliana* leaves. (E) The protein complexes penetrated through the cell wall and cell membrane and localized to the cytoplasm or were imported into the peroxisome or nucleus, depending on the fused organelle-targeting peptide.

Previous studies demonstrated that CPP-fused to a polycationic peptide [an alternate copolymer of lysine and histidine, (KH)_9_], which was termed as fusion peptides, is an more efficient nucleic acid carrier compared with that of the CPP alone in intact plants [21, 22]. By using the fusion peptides, the negatively charged cargo preferentially interacts with the polycationic peptide through ionic interactions, whereas the CPP interacts with fewer cargo molecules and is preferentially present on the surface of peptide-cargo complexes. The more presence of CPP at the surface of the complexes leads to a higher efficiency in gene delivery [23]. In the current study, based on the concept of fusion peptide, two protein delivery carriers, (BP100)_2_K_8_ and BP100(KH)_9_, were designed by fusing a CPP, i.e., BP100 or BP100 dimer, and a polycationic peptide, i.e., alternate copolymer of lysine and histidine, (KH)_9_ or eight consecutive lysine residues, K_8_ (Fig 1). The poly-lysine is known to destabilize cell-membrane via electrostatic interaction between the protonated amine and the negatively charged cell membrane [24]. On the other hand, the copolymer of lysine and histidine has the ability to promote the buffering effect on the pre-lysomal vesicle [25]. In current study, we assumed that the polycationic peptides [(KH)_9_ and K_8_] also play an important role in the electrostatic interaction with negatively charged protein cargo, similarly to the ionic interaction with plasmid DNA (pDNA) as described in previous studies [21, 26, 27]. BP100, originally used as an antimicrobial peptide against plant pathogens [28], has been exploited as an efficient CPP in intact plant cells [15, 21, 26, 27]. In previous studies, dimeric CPP has shown significantly higher gene transfection efficiency in comparison to monomeric CPP [29]. With two copies of CPP at the N-terminus, the second CPP may act as a linker preventing the polycationic peptide at the C-terminus from affecting the α-helical conformation of the first CPP, which is a crucial structure for cellular internalization [30]. Based on this concept, we designed a new fusion peptide, (BP100)_2_K_8_ containing the BP100 dimer at the N-terminus and a poly-lysine, K_8_, at the C-terminus to maintain the α-helical conformation and cell-penetrating properties of the first BP100 at the N-terminus. Other than (BP100)_2_K_8,_ we also aimed to evaluate the protein delivery efficiency of BP100(KH)_9_, an efficient nuclei acid carrier described in previous studies [21, 26, 27].

To elucidate the intracellular protein delivery efficiencies of (BP100)_2_K_8_ and BP100(KH)_9_, negatively charged proteins with various molecular weights, including fluorescent protein (Citrine) (27 kDa), bovine serum albumin (BSA) (66 kDa) and alcohol dehydrogenase (ADH) (150 kDa), were used as protein cargoes. In addition, two proteins conjugated with organelle-targeting peptides, Citrine-nuclear localization signal (NLS) and Citrine-peroxisomal targeting signal (PTS), were used to investigate whether the fusion peptide interfered with the function of organelle-targeting peptides. The negatively charged proteins interacted with cationic carrier peptide via electrostatic interaction to form peptide/protein complexes that can be delivered into the cells. This study represents the first attempt to deliver exogenous proteins directly into intact plants via fusion peptides, which represents an initial step toward the goal of using direct protein delivery approaches in more plant biotechnology applications.

## Results

### Characterization of Peptide/BSA Complexes

**Fig 2:**
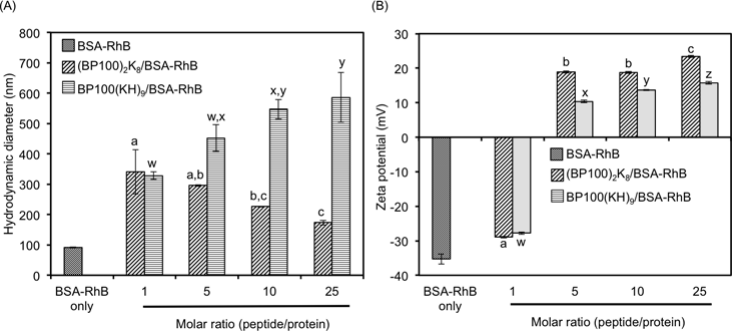
Characterization of peptide/BSA-RhB complex formation. (A) Hydrodynamic diameters and (B) zeta potentials of the peptide/BSA-RhB complexes prepared at peptide/protein molar ratios ranging from 1–25. All data are expressed as the means ± S.D. from triplicate tests; the mean data labeled with different letters are significantly different (Tukey’s HSD test, *p* < 0.05).

**Fig 3:**
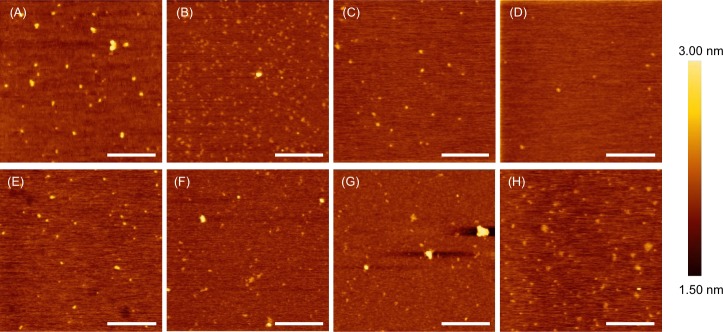
Morphologies of the peptide/BSA-RhB complexes as observed under AFM. The (BP100)_2_K_8_/BSA-RhB complexes were prepared at peptide/protein molar ratios of 1 (A), 5 (B), 10 (C) and 25 (d). The BP100(KH)_9_/BSA-RhB complexes were prepared at peptide/protein molar ratios of 1 (E), 5 (F), 10 (G) and 25 (H). Scale bars: 500 nm.

The cationic BP100(KH)_9_ and (BP100)_2_K_8_ peptides were first tested for their ability to form ionic complexes with negatively charged proteins via electrostatic interactions. The BSA (66 kDa), a negatively charged protein, was selected as a model protein to investigate ionic complex formation. Prior to complex formation, the BSA was conjugated with Rhodamine B isothiocyanate (RhB) to prepare the BSA-RhB conjugate (BSA-RhB), which is fluorescent and can be visualized in gels or under fluorescence microscopy. Sodium dodecyl sulfate-polyacrylamide gel electrophoresis (SDS-PAGE) of BSA-RhB was performed to confirm the conjugation of RhB to BSA (S1 Fig). Both cationic BP100(KH)_9_ and (BP100)_2_K_8_ were individually mixed with the negatively charged BSA-RhB conjugate at various peptide/protein molar ratios (the protein molarity remained constant, and the peptide molarity increased from 1 to 25) to form the ionic peptide/protein complexes (BP100)_2_K_8_/BSA-RhB and BP100(KH)_9_/BSA-RhB. The hydrodynamic diameters (diameters in solution), zeta potentials (surface charge) and morphologies of the BSA-RhB conjugate and peptide/protein complexes were characterized by Zetasizer Nano-ZS (Fig 2) and atomic force microscopy (AFM) (Fig 3). The hydrodynamic diameter of BSA-RhB was 92 ± 2 nm. As the peptide/protein molar ratios increased, the average hydrodynamic diameters of (BP100)_2_K_8_/BSA-RhB decreased from 341 ± 73 nm to 173 ± 8 nm (Fig 2A and S1 Table). In contrast, the average hydrodynamic diameters of the BP100(KH)_9_/BSA-RhB complexes increased from 329 ± 12 nm to 586 ± 82 nm as peptide/protein molar ratios increased (Fig 2A and S2 Table). The polydispersity indexes (PDI) of all complexes are listed in S1 and S2 Tables. The (BP100)_2_K_8_/BSA-RhB complexes prepared at molar ratios of 5 and 10 and the BP100(KH)_9_ complexes prepared at a molar ratio of 1 exhibited homogeneous globular morphologies (Fig 3B–3D and S2 Fig). BSA-RhB had a negative surface charge of -35.3 ± 1.5 mV (Fig 2B). As the peptide/protein molar ratios increased, the zeta potential of both (BP100)_2_K_8_/BSA-RhB and BP100(KH)_9_/BSA-RhB complexes increased from negative to positive (Fig 2B, S1 and S2 Tables). This result indicates that the surface of BSA-RhB was covered by cationic fusion peptides, resulting in ionic complex formation and increased surface charge.

### Quantification of BSA Delivery Efficiency

**Fig 4:**
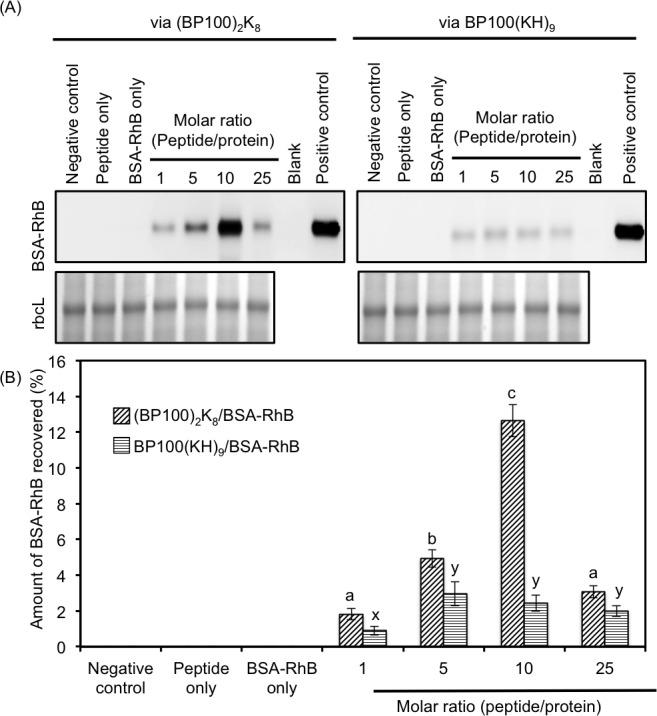
Efficiency of BSA-RhB delivery by a (BP100)_2_K_8_ carrier or a BP100(KH)_9_ carrier. (A) SDS-PAGE of the total crude proteins extracted from the transgenic YFP *A*. *thaliana* leaves at 6 h post-infiltration with (BP100)_2_K_8_/BSA-RhB complexes and BP100(KH)_9_/BSA-RhB complexes prepared at peptide/protein molar ratios ranging from 1 to 25. Two micrograms of BSA-RhB were used for each group. The fluorescent band of BSA-RhB was detected with a luminescence image analyzer while the rbcL band was detected by Coomassie-blue staining. The negative control was the total crude protein extracted from the non-infiltrated transgenic YFP *A*. *thaliana* leaf. The positive control was BSA-RhB, which was loaded directly onto the SDS gel as the size control. (B) The bar graph shows the total amount (%) of BSA-RhB extracted from the transgenic YFP *A*. *thaliana* leaves after infiltration, based on the intensity of fluorescent BSA-RhB band normalized to Coomassie-blue stained rbcL band. All data shown are expressed as the means ± S.D. of triplicate tests; the mean data labeled with different letters are significantly different (Tukey’s HSD test, *p* < 0.05).

After the characterization of the peptide/protein complexes, we sought to evaluate the BSA delivery efficiencies of the various peptide/protein molar ratios and of the different types of fusion peptides. The BSA delivery efficiency was quantified based on the amount of intact BSA-RhB recovered from the infiltrated leaves. The transgenic *A*. *thaliana* expressing yellow fluorescent protein (YFP *A*. *thaliana*) was infiltrated with the fusion peptides alone, BSA-RhB alone, the (BP100)_2_K_8_/BSA-RhB complexes or the BP100(KH)_9_/BSA-RhB complexes. Various peptide/protein molar ratios were tested, and the total proteins from a whole leaf were extracted at 6 h post-infiltration. The extracted protein lysates were analyzed with SDS-PAGE, and BSA-RhB (66 kDa) was excited and visualized with a luminescence image analyzer. As shown in Fig 4A, BSA-RhB was present only when the fusion peptide was used as a carrier. Because BSA-RhB was infiltrated without a carrier peptide, no BSA-RhB signal was detected in the gel, indicating that the protein did not enter and was not retained in the cells. The BSA-RhB standard protein was run in the gel as a positive control, whereas fusion peptide infiltration and non-infiltrated YFP *A*. *thaliana* were used as negative controls to provide a clean background. The differences in fluorescent intensity were observed among the different fusion peptides and also among the different peptide/protein molar ratios (Fig 4A). The intensity of BSA-RhB was normalized to the intensity of the Rubisco large subunit (rbcL), the most abundant protein in plant cells. The normalized amount of recovered BSA-RhB was compared among the different fusion peptides and among various peptide/protein molar ratios (Fig 4B). (BP100)_2_K_8_ showed better protein delivery than BP100(KH)_9_ at all of the tested peptide/protein molar ratios. Among all of the peptide/protein molar ratios containing (BP100)_2_K_8_, the peptide/protein molar ratio of 10 exhibited the highest amount of recovered protein (12.6%), indicating the highest protein delivery efficiency.

### Intracellular Delivery of BSA and Time Course Evaluation

**Fig 5:**
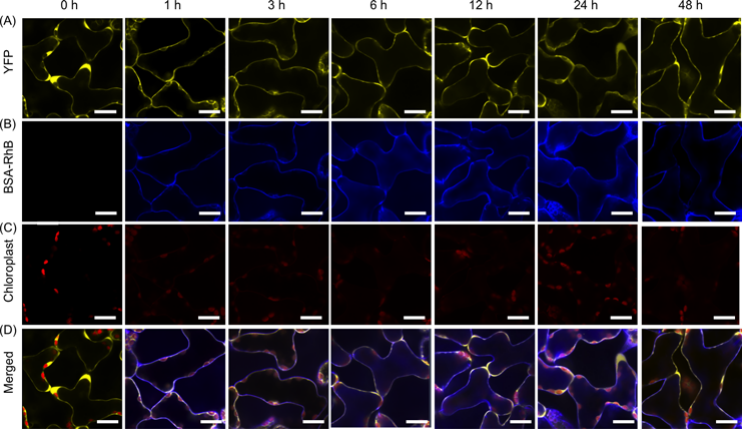
Time-course analyses of BSA-RhB delivery by the (BP100)_2_K_8_ carrier at a peptide/protein molar ratio of 10. Transgenic YFP *A*. *thaliana* leaves infiltrated with (BP100)_2_K_8_/BSA-RhB complexes were observed at various time points (0, 1, 3, 6, 12, 24 and 48 h) by CLSM. (A) YFP fluorescence in the cytosol of transgenic YFP *A*. *thaliana* leaf, (B) RhB fluorescence from the infiltrated BSA-RhB conjugates, (C) autofluorescence of the plastid (chloroplast) of sponge cells, (D) overlay of the four images (a-c). Scale bars: 20 μm.

We sought to determine the intracellular delivery of the BSA-RhB and then evaluated the time course effect. The optimum conditions of (BP100)_2_K_8_ with a peptide/protein molar ratio of 10 were used. The (BP100)_2_K_8_/BSA-RhB complexes were infiltrated into one YFP *A*. *thaliana* leaf for 6 h, and the fluorescence images were obtained by confocal laser scanning microscopy (CLSM) as shown in Fig 5. The cytosolic and nuclear expression of YFP fluorescence in the transgenic YFP *A*. *thaliana* [31] was used to distinguish the intracellular space from the extracellular space. At 1 h and 3 h post-infiltration, BSA-RhB fluorescence was detected predominantly on the extracellular surface of the cells and was subsequently shown to co-localize with the YFP fluorescence from 6 to 48 h post-infiltration, indicating that BSA-RhB was located in the intracellular space (Fig 5). In addition to its localization inside the cytosol, the BSA-RhB signal was also found in the vacuole from 6 to 24 h post-infiltration. At 48 h post-infiltration, faint BSA-RhB fluorescence was observed inside the cells. Negative controls of infiltration consisting of BSA-RhB alone and carrier peptide alone were analyzed in parallel, and no BSA-RhB was detected throughout the leaves (S3A–S3B Fig). The infiltration of the peptide/protein complexes was also assessed in a wild-type *A*. *thaliana* leaf under the same conditions as in the YFP *A*. *thaliana* leaf. RhB fluorescence was observed inside the cells of the infiltrated wild-type *A*. *thaliana* leaf (S3C Fig). Thus, the possibility of false fluorescence from the YFP can be ruled out.

### Intracellular Delivery of High-Molecular-Weight of Protein (150 kDa)

**Fig 6:**
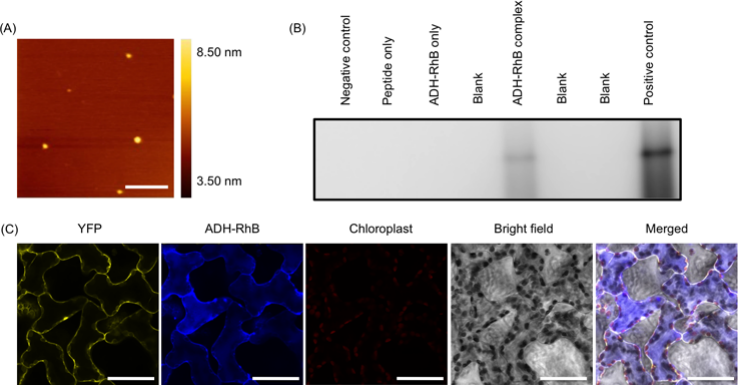
Characterization of (BP100)_2_K_8_/ADH-RhB complex formation and intracellular delivery efficiency. The (BP100)_2_K_8_/ADH-RhB complexes were prepared at a peptide/protein molar ratio of 10. (A) AFM image of the complexes. Scale bars: 500 nm. (B) Native PAGE of the total crude proteins extracted from the transgenic YFP *A*. *thaliana* leaves at 6 h post-infiltration. The fluorescent band of ADH-RhB was detected with a luminescent image analyzer. The negative control was the total crude protein extracted from the non-infiltrated transgenic YFP *A*. *thaliana* leaf. The positive control was ADH-RhB, which was loaded directly onto the gel as the size control. (C) CLSM observation of the leaves at 6 h post-infiltration. Scale bars: 50 μm.

We demonstrated that the fusion peptide was capable of delivering BSA-RhB into the intact plants via infiltration. Subsequently, we tested whether the fusion peptide could mediate the intracellular delivery of proteins with high molecular weights at the optimum conditions used for BSA delivery, which included using (BP100)_2_K_8_ as the carrier peptide and a peptide/protein molar ratio of 10 at 6 h post-infiltration, The ADH (150 kDa) was selected as a model protein. The negatively charged ADH protein was conjugated to RhB to form a fluorescent ADH-RhB conjugate, and fluorophore labeling was confirmed by native PAGE of ADH-RhB (S4 Fig). ADH-RhB exhibited a negative zeta potential (-43.1 ± 1.1 mV) and a hydrodynamic diameter of 168 ± 7 nm. The ADH-RhB was mixed with (BP100)_2_K_8_ at a peptide/protein molar ratio of 10 to form the peptide/protein complexes, and the complex formation was characterized. The average hydrodynamic diameter of the (BP100)_2_K_8_/ADH-RhB complex was 308 ± 53 nm, the PDI value was 0.21 ± 0.07, and the surface charge was 11.1 ± 1.9 mV. The increase in zeta potential from -43.1 to 11.1 mV indicated that the surface of ADH-RhB was covered by cationic (BP100)_2_K_8_ in ionic complex. The (BP100)_2_K_8_/ADH-RhB complex showed homogeneous globular morphology (Fig 6A and S5A Fig).

Subsequently, the complex was infiltrated into a YFP *A*. *thaliana* leaf for 6 h fluorescence images were obtained by CLSM. The ADH-RhBs were detected inside the cytosol and vacuoles of all cells at 6 h post-infiltration (Fig 6C), similarly to the results observed after BSA delivery. In addition, the ADH-RhB signal was also detected in the wild-type *A*. *thaliana* leaf, confirming that the fluorescence was from ADH-RhB (S5B Fig). ADH-RhB was successfully recovered from the leaf infiltrated with the (BP100)_2_K_8_/ADH-RhB complexes, as detected with native PAGE (Fig 6B). The ADH-RhB standard protein was used as a positive protein control. The ADH-RhB signal was not detected in the leaf infiltrated with ADH-RhB alone (S5C Fig). This result further supports that the protein is unable to translocate into the cells without any carrier peptide. The successful delivery of ADH-RhB indicates that the fusion-peptide-mediated protein delivery system is feasible for proteins with relatively high molecular weights.

### Intracellular Localization of Proteins with Organelle-Targeting Peptides

**Fig 7:**
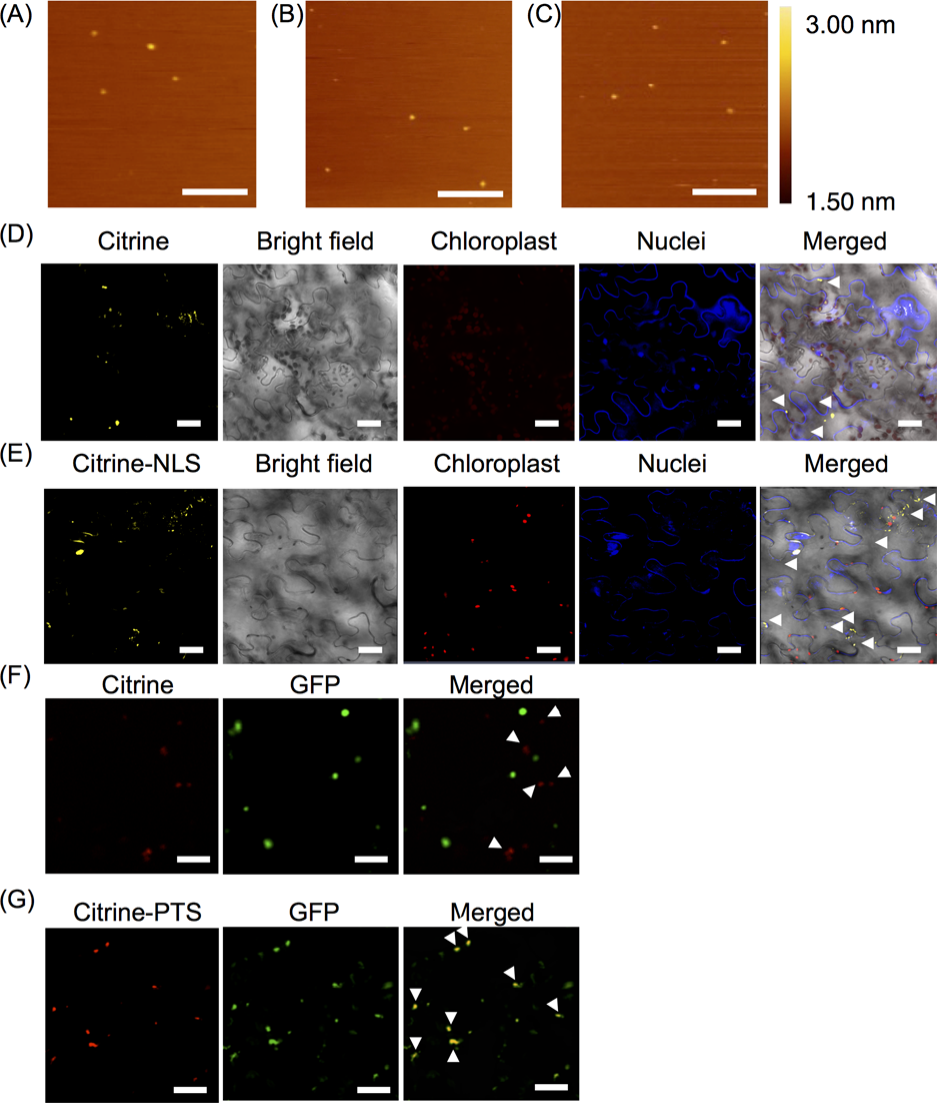
Characterization of (BP100)_2_K_8_-mediated complex formation with Citrine, Citrine-NLS or Citrine-PTS and the intracellular delivery of proteins. The peptide/protein complexes were prepared at a peptide/protein molar ratio of 10. (a-c) AFM images of (BP100)_2_K_8_/Citrine complexes (A), (BP100)_2_K_8_/Citrine-NLS complexes (B) and (BP100)_2_K_8_/Citrine-PTS complexes (C). Scale bars: 500 nm. (d-g) Protein localization was observed using CLSM. Scale bars: 20 μm. The arrowheads in the merged images indicate protein localization. The leaves of wild type *A*. *thaliana* were infiltrated with (BP100)_2_K_8_/Citrine complexes (as a negative control) (D) and (BP100)_2_K_8_/Citrine-NLS complexes (E). The nuclei were stained with 4’,6-diamidino-2-phenylindole (DAPI). The leaves of *A*. *thaliana* expressing GFP-PTS were infiltrated with (BP100)_2_K_8_/Citrine complexes (as a negative control) (F) and (BP100)_2_K_8_/Citrine-PTS complexes (G). The peroxisome was indicated by GFP fluorescence.

Finally, we investigated whether the carrier peptide, (BP100)_2_K_8_, could deliver the protein that conjugated to an organelle-targeting peptide, because the carrier peptide may interact with the organelle-targeting peptide and interfere with the function of the organelle-targeting peptide. There is a possibility that the function of the organelle-targeting peptide that conjugated to the protein of interest is hindered after labeling with extrinsic fluorescent dye. In order to evaluate the organelle-targeting function that solely affected by the use of carrier peptide, (BP100)_2_K_8_, an intrinsically fluorescent protein (Citrine) instead of fluorescently labeled-protein (BSA-RhB or ADH-RhB) was used. Citrine was conjugated to a NLS for nucleus targeting and PTS for peroxisome targeting, respectively. Citrine without any organelle-targeting peptide was used as a negative control. (BP100)_2_K_8_ was mixed individually with Citrine, Citrine-NLS and Citrine-PTS at a peptide/protein molar ratio of 10 based on the optimum conditions of BSA delivery. Three types of Citrine proteins and the Citrine complexes of (BP100)_2_K_8_ were characterized with respect to their hydrodynamic diameters, zeta potentials and morphologies. The three types of Citrine proteins exhibited negative zeta potentials and hydrodynamic diameters of approximately 200 nm (S3 Table). The positively charged complexes of Citrine, Citrine-NLS and Citrine-PTS were formed with sizes ranging from 218 ± 13 nm to 263 ± 7 nm (S4 Table). Based on AFM observations (Fig 7A–7C) and the size distribution graph (S6 Fig), all of the complexes formed homogeneous globular complexes.

The localization of Citrine-NLS and Citrine-PTS in the targeted organelle was observed by CLSM. The (BP100)_2_K_8_/Citrine complexes were infiltrated into the wild-type *A*. *thaliana* and transgenic *A*. *thaliana* expressing the green fluorescent protein (GFP)-peroxisomal targeting signal (GFP-PTS) as a negative control. The (BP100)_2_K_8_/Citrine-NLS complexes were infiltrated into the wild-type *A*. *thaliana*, and the (BP100)_2_K_8_/Citrine-PTS complex was infiltrated into the transgenic *A*. *thaliana* expressing GFP-PTS. Citrine without an organelle targeting-peptide was localized inside the cells with no specified compartment (Fig 7D and 7F). In contrast, Citrine-NLS accumulated in a defined area that overlapped with DAPI staining, a nuclear indicator. However, the localization of Citrine-NLS inside the nucleus was detected only at 72 h post-infiltration (Fig 7E). The leaf peroxisomes of *A*. *thaliana* expressing GFP-PTS appeared as green spherical dots due to the presence of GFP [32] (Fig 7F and 7G). Citrine-PTS was localized in the peroxisome, as indicated by the overlapping fluorescence of Citrine and GFP (Fig 7G). Citrine-PTS was successfully delivered into the peroxisome at 18 h post-infiltration. The infiltration of Citrine-PTS without a carrier peptide was carried out in parallel, and no Citrine-PTS was detected throughout the leaf (S7 Fig), indicating that Citrine with an organelle-targeting peptide can enter the cell only via the carrier peptide. Considering these results, the function of organelle-targeting peptides was not hampered by the fusion peptide (BP100)_2_K_8,_ as the proteins were successfully delivered into the targeted organelles.

## Discussion

To date, although many *in vitro* studies have been reported the use of direct protein delivery systems into plant cells in corn root tip cells, tobacco BY-2 cells, microspore cultures, and onion epidermal cells, these studies have used the strategy only in cellular-level assays [12–18]. To the best of our knowledge, this study is the first to demonstrate a successful direct protein delivery system in intact plants by using fusion peptides (a combination of polycationic peptide and cell penetrating peptide). Several properties of this direct protein delivery strategy are demonstrated. First, the delivery system is feasible with negatively charged proteins with various molecular weights ranging from 27 kDa to 150 kDa. Second, the fusion peptide does not interfere with the function of an organelle-targeting peptide when conjugated to the protein cargo. Finally, protein delivery is rapid, thus allowing the delivered protein to enter the cells as early as 6 h post-infiltration.

Our non-covalent protein delivery strategy has shown some advantages over the covalent protein delivery strategy that are commonly used (S5 Table). First, our peptide/protein complex preparation is simple and rapid. The complex can be formed within 30 min by direct mixing of peptide and protein of interest. Meanwhile, the covalent-linking strategy is time-consuming and laborious as it requires prior chemical covalent bonding or molecular cloning followed by protein expression. Second, our carrier peptide can be a versatile tool with various protein cargoes of opposite charge to form peptide/protein complexes. In contrast, specific customization for chemical cross-linking or plasmid construction is required for each peptide-fusion protein. Lastly, the biological activities of our protein cargoes are preserved. The detection of fluorescent signal of proteins inside the cells confirms the structural integrity of the protein delivery, which most likely reflects a preserved biological activity (Fig 5, Fig 6C, Fig 7D–7G). On the other hand, protein may lose its biological activity after protein is covalently fused to an additional sequence [20]. The peptide/protein complexes are formed via electrostatic interaction and the interaction does not affect the protein function significantly. Thus, the protein cargoes can be delivered in biologically active form. In the case of peptide-fusion protein, synthetic linkage may affect the conformation of protein and interfere the biological activity of protein cargoes. The side-by-side comparison between the two methodologies implies that our non-covalent strategy involving the electrostatic interaction can be applied in a robust protein delivery system without any interference on biological activity of protein cargoes.

BSA was selected as model protein for the optimization of protein delivery conditions, such as type of carrier peptide, peptide/protein molar ratio and time course effect. The result of BSA delivery showed that (BP100)_2_K_8_ exhibited a better delivery ability than that of BP100(KH)_9,_ suggesting that the BP100 dimer may enhance the cell-penetrating power of the carrier peptide, greatly facilitating the intracellular delivery of proteins. In order to test whether the BSA delivery conditions could be generalized for the delivery of diverse proteins, the cell penetrating ability of (BP100)_2_K_8_ with peptide/protein molar ratio of 10 was further tested for the delivery of ADH-RhB and the three versions of Citrine (Citrine, Citrine-NLS and Citrine-PTS). The results clearly showed that (BP100)_2_K_8_ with peptide/protein molar ratio of 10 is able to deliver proteins into the cells, while maintaining the function of organelle-targeting peptide.

The size range, degree of homogeneity, and the positive charges of the peptide/cargo complex are critical to an efficient delivery because they facilitate different cellular uptake mechanisms [33–37]. For example, cationic peptide/protein complexes of 200 to 1000 nm can enter cells via lipid raft-dependent macropinocytosis [15, 16, 36–38]. Moreover, positively charged protein complexes have been postulated to interact with the negatively charged heparan sulfate proteoglycans on the surface of the cell membrane, thus subsequently triggering the formation of membrane protrusions and endocytosis [13].

Based on the time course analysis, BSA-RhB was detected inside vacuole from 6 to 24 h post-infiltration. To control the protein turnover inside the cell, cytoplasmic proteins were imported into the vacuole for proteolysis. It was likely that the BSA-RhB located inside cytosol was imported into the vacuole together with cytoplasmic proteins for non-specific proteolysis [39, 40]. As time proceeds, therefore, most of the delivered BSA-RhB was imported into the vacuole for proteolysis. At 48 h post-infiltration, very low amount of protein was detected inside the cytosol, because the BSA-RhB inside the vacuole was degraded, as indicated by the loss of BSA-RhB signal.

The highest yield of proteins that we could recover from the infiltrated leaf was about 12.6%. This result indicated that most of the proteins failed to enter the cells. After the peptide/protein complex was infiltrated into the leaf (Fig 1D), the peptide/complexes were firstly distributed at the extracellular space of the plant cells. The plant extracellular space contains proteases that protect the plant against pathogen attack. As a defense mechanism, the proteases are likely to degrade any exogenous proteins or virulence factors at extracellular space [41, 42]. Hence, there is possibility that some of the peptide/protein complexes are degraded by the extracellular protease before they translocate across the cell membrane, resulting in low protein delivery efficiency.

Based on CLSM, we demonstrated that BSA-RhB, ADH-RhB and Citrine could be delivered intracellularly at high efficiencies, whereas the delivery efficiencies of Citrine-NLS and Citrine-PTS into the nucleus and the peroxisome, respectively, were relatively lower. One possible explanation for this result is that in some case, the carrier peptide did not dissociate from the protein cargo, resulting in a structurally hindered NLS or PTS. The structurally hindered NLS or PTS would not be recognized by importin or peroxin, respectively [43, 44], thus resulting in slightly lower delivery efficiencies to the target organelles.

In this study, we have performed comprehensive methods to ensure the successful delivery of proteins into cytosol or nucleus or peroxisomes, because there are no alternative and established methods to confirm the protein delivery. As shown in Figs 4A and 6B, BSA-RhB and ADH-RhB signals were only detected when the carrier peptide was used, indicating that the BSA-RhB and ADH-RhB were only delivered into the cells via carrier peptide and retained inside the cells. In the CLSM experiment, the cytosolic and nuclear expression of YFP fluorescence in the transgenic YFP *A*. *thaliana* was used to distinguish the intracellular space from the extracellular space and to confirm the intracellular localization of BSA-RhB and ADH-RhB. Both BSA-RhB signal (Fig 5) and ADH-RhB signal (Fig 6) were detected in the cytosol, based on the overlapping signal of YFP and RhB. The BSA-RhB and ADH-RhB signals were not detected in the negative control experiment using either BSA-RhB alone (S3A Fig) or ADH-RhB alone (S5C Fig). This result further supports the intracellular delivery of BSA-RhB and ADH-RhB via our deigned carrier peptide. In the organelle-targeting study, we performed CLSM to detect the localization of protein in the targeted organelle. The Citrine-NLS was co-localized with DAPI in nucleus (Fig 7E), while the Citrine-PTS was co-localized with GFP signal in peroxisome (Fig 7G). On the other hand, Citrine without any organelle-targeting peptide was not detected in the targeted organelle (Fig 7D and 7F), indicating that the detection of Citrine-PTS and Citrine-NLS in targeted organelles was not from false fluorescence. Thus, the comprehensive methods we used are appropriate enough to evaluate the protein delivery system.

In conclusion, we successfully demonstrated a feasible fusion-mediated protein delivery system in intact plants that enables plant engineering without the risk of introducing random integration of exogenous DNA into the plant nuclear genome or organelle genome. Given the potent fusion-peptide mediated delivery of various types of anionic proteins, we speculated that CRISPR-Cas9:sgRNA complexes, with anionic properties, might also be delivered into intact plant to perform targeted genome editing using this approach. Our approach could ensure that after genome editing, no unexpected genes would be left in the target genome. The feasibility of our peptide-based delivery in genome editing would be explored in the near future. Hence, the fusion-peptide-mediated protein delivery system is a powerful tool for the safe, simple and rapid modification of plants in crop breeding and plant biology research.

## Materials and Methods

### Plant Growth Condition

The transgenic YFP (yellow fluorescent protein) *A*. *thaliana* was generated by the *Agrobacterium tumefaciens* [strain GV3101 (pMP90)]-mediated transformation of binary vector containing cauliflower mosaic virus 35S promoter and In-YFP gene into wild-type *A*. *thaliana* (Columbia) [31]. The seeds of both wild-type *A*. *thaliana* and transgenic YFP *A*. *thaliana* were sowed in pots with planting medium containing a mixture of soil (Pro-Mix; Premier Tech Ltd, Quebec, Canada) and vermiculite (Vskakou, Tokyo, Japan) in a ratio of 2:1. The transgenic *A*. *thaliana* expressing GFP-PTS was generated by the *A*. *tumefaciens* [strain EHA101]-mediated transformation of pMAT137 containing sGFP gene into wild-type *A*. *thaliana* (Columbia) [32]. The seeds were sowed on 1% agar containing Murashige and Skoog (MS) medium with kanamycin. The kanamycin-resistant seedlings were germinated and maintained on MS medium for 1 week. After 1 week, the plants were transferred to the pots with planting medium containing a mixture of soil and vermiculite in a ratio of 2:1. All the *A*. *thaliana* were grown under long-day conditions (16 h light/8 h dark) at 21°C in a plant incubator (Biotron NK System, Osaka, Japan).

### Peptide Synthesis

(BP100)_2_K8 (KKLFKKILKYLKKLFKKILKYLKKKKKKKK, theoretical pI/Mw: 10.75/3851.13 Da) and BP100(KH)_9_ (KKLFKKILKYLKHKHKHKHKHKHKHKHKH, theoretical pI/Mw: 10.81/ 3809.71 Da) were synthesized using standard 9-fluorenylmethoxycarbonyl solid-phase peptide synthesis, followed by the purification using high-performance liquid chromatography [45]. The molecular weights of the peptides were determined by matrix-assisted laser desorption/ionization–time-of-flight mass spectrometry.

### Preparation of Rhodamine B Isothiocyanate Labeled Proteins

Rhodamine B isothiocyanate (RhB) (Mw: 536.08 g/mol), BSA and ADH from *Saccharomyces cerevisiae* were purchased from Sigma-Aldrich (St. Louis, MO, USA). First, 10.0 g/L of BSA or ADH solution was prepared by dissolving 10 mg of protein powder in 1 mL of sodium carbonate solution (0.1 M, pH 9.0), and 10.0 g/L of RhB solution was prepared by dissolving 1 mg of RhB powder in 100 μL of dimethyl sulfoxide. Then, RhB was conjugated to the protein by drop wise addition of the RhB solution to the protein solution under gentle stirring, followed by incubation at 4°C overnight with continuous stirring. The free RhB dye was removed from the BSA-RhB conjugate or the ADH-RhB conjugate by gel filtration chromatography using a Sephadex G-25 column (Sigma-Aldrich, St. Louis, MO, USA) at 25°C. The concentration of the purified BSA-RhB conjugate or ADH-RhB conjugate was measured by UV−vis spectrophotometry at 280 nm and 555 nm. The degree of labeling was calculated using the following formula: (OD_555 nm_ of RhB × MW of protein)/ (protein concentration in g/L × molar extinction coefficient of RhB). The molar extinction coefficient of RhB is 106,000 M^-1^ cm^-1^_._ In this study, on average, 4 molecules of RhB were conjugated to one molecule of BSA, and 6 molecules of RhB were conjugated to one molecule of ADH.

### Construction of Plasmids for Expression of Citrine, Citrine-NLS and Citrine-PTS

To construct plasmids for the cell-free synthesis of Citrine, Citrine-NLS and Citrine-PTS proteins, the gateway cloning system (Invitrogen, Carlsbad, CA, USA) was used according to the manufacturer’s instructions. The Citrine gene was amplified using PCR with the following primers: 5’-GGGGACAAGTTTGTACAAAAAAGCAGGCTCCATGGTGAGCAAGGGCGAG-3’ and 5’-GGGGACCACTTTGTACAAGAAAGCTGGGTCTCACTTGTACAGCTCGTCC-3’. The PCR-amplified DNA fragment was cloned into the pDONR207 donor vector (Invitrogen) by using the gateway BP reaction, and the resulting plasmid was named pDONR207-Citrine. For Citrine-NLS and Citrine-PTS, pDONR207-Citrine-NLS and pDONR207-Citrine-PTS were used (Ogasawara et al., 2013). The plasmids contained the sequences for NLS [Pro-Pro-Lys-Lys-Lys-Arg-Lys-Val] and PTS1 [Ser-Lys-Leu], respectively. The pDONR207-Citrine, pDONR207-Citrine-NLS and pDONR207-Citrine-PTS plasmids were mixed with the pDEST17 destination vector (T7 promoter and N-terminal 6×His-tag) (Invitrogen), and LR reactions were performed to generate pDEST17-Citrine, pDEST17-Citrine-NLS and pDEST17-Citrine-PTS.

### Cell-Free Synthesis of Citrine, Citrine-NLS and Citrine-PTS

The dialysis-mode cell-free protein synthesis method was used in this study [46, 47]. Briefly, the internal solution (9 mL) consisted of substrates, buffers, plasmid and enzymes required for transcription and translation. The solution contained 55 mM HEPES-KOH buffer, pH 7.5 (containing 1.7 mM dithiothreitol); 68 μM L(–)-5-formyl-5,6,7,8-tetrahydrofolic acid); 0.05% sodium azide; 4.0% polyethyleneglycol (average molecular weight 8,000 g/mL); 210 mM potassium glutamate; 27.5 mM ammonium acetate; 10.7 mM magnesium acetate; 2.7 mL of S30 extract; 1.2 mM adenosine-5'-triphosphate (pH 7.0); 0.8 mM each cytidine triphosphate (pH 7.0), guanosine-5'-triphosphate (pH 7.0), and uridine-5'-triphosphate (pH 7.0); 80 mM creatine phosphate; 0.64 mM 3',5'-cyclic adenosine monophosphate; 1.0 mM each of the 20 amino acids; 175 μg/mL *E*. *coli* total tRNA; plasmid constructs (pDEST17-Citrine plasmid or pDEST17-Citrine-NLS plasmid or pDEST17-Citrine-PTS plasmid); 250 μg/mL creatine kinase; and 93 μg/mL T7 RNA polymerase. The S30 extract was prepared from the *E*. *coli* BL21 codon-plus RIL strain (Stratagene, La Jolla, CA), as previously described [48]. Cell-free giant-scale dialysis using a dialysis membrane with a molecular weight cutoff of 15 kDa (Pierce, Rockford, IL) was performed, and 9 mL of the internal solution was dialyzed against 90 mL of the external solution. The reaction mixture was incubated at 30°C for 16 h with shaking.

### Purification of Citrine, Citrine-NLS and Citrine-PTS

Nine milliliters of the internal solution with the tagged protein (Citrine or Citrine-NLS or Citrine-PTS) was purified via AKTA Express (GE Healthcare, Little Chalfont, UK). The internal solution was centrifuged at 3,000 × *g* for 30 min, and the supernatant was mixed with 18 mL of buffer A [20 mM Tris-HCl buffer (pH 8.0) containing 300 mM sodium chloride, 20 mM imidazole, and 1 mM Tris (2-carboxyethyl) phosphine hydrochloride (TCEP, Hampton Research, Aliso Viejo, CA)]. The protein solution (Citrine or Citrine-NLS or Citrine-PTS) was loaded onto 5 mL of HisTrap (GE Healthcare, Little Chalfont, UK), and the resins were washed with buffer A. The proteins (Citrine or Citrine-NLS or Citrine-PTS) were eluted with buffer B (20 mM Tris-HCl buffer [pH 8.0] containing 300 mM sodium chloride, 500 mM imidazole, and 1 mM TCEP).

### Preparation and Characterization of the Peptide/Protein Complexes at Various Peptide/Protein Molar Ratios

To prepare the peptide/protein complexes, 1.0 g/L peptide [(BP100)_2_K_8_ or BP100(KH)_9_] was mixed with 2 μg of protein (BSA-RhB conjugates, ADH-RhB conjugates, Citrine, Citrine-NLS or Citrine-PTS) at various peptide/protein molar ratios (1, 5, 10 and 25); this was followed by dilution with autoclaved Milli-Q water to a final volume of 100 μL. Then, 10 μL of solution was aliquoted and further diluted to a total volume of 100 μL. The average hydrodynamic diameter of the complexes was measured by dynamic light scattering (DLS) using a Zetasizer Nano-ZS (Malvern Instruments, Ltd., Worcestershire, UK). The polydispersity index (PDI) was determined with the Zetasizer software ver. 6.20 using a 633 nm He−Ne laser at 25°C with a backscatter detection angle of 173°. Then, the samples were further diluted to a total volume of 750 μL with autoclaved Milli-Q water and were analyzed with Laser Doppler Microelectrophoresis using a Zetasizer Nano-ZS for zeta potential. The zeta potential and zeta deviation of the samples were measured three times, and the average data were obtained using Zetasizer software ver. 6.20. For morphology characterization by atomic force microscopy (AFM), 10 μL of the peptide/protein complex solutions (1.0 mg/L) prepared at various peptide/protein molar ratios were dropped onto freshly cleaved mica and allowed to absorb onto the mica substrate for 30 seconds [49]. Then, the samples were rinsed thoroughly with autoclaved Milli-Q water to remove all of the buffer components. After the residual water was drained from the mica surface, the mica was air-dried at room temperature overnight. The samples were observed in air at room temperature using a silicon cantilever with a spring constant of 1.7 N/m (Type: SI-DF3) in tapping mode AFM (AFM5300E, Hitachi High-Tech Science Corporation, Japan).

### Infiltration into Leaves with Peptide/Protein Complexes

Approximately 100 μL of peptide/protein complex solution with various peptide/protein molar ratios (1, 5, 10 and 25) was infiltrated directly into the leaves of *A*. *thaliana* by using a syringe without a needle (Fig 1). The leaves of *A*. *thaliana* expressing YFP were infiltrated with peptide/BSA-RhB complexes or peptide/ADH-RhB complexes; the wild-type *A*. *thaliana* leaves were infiltrated with peptide/Citrine complexes or peptide/Citrine-NLS complexes; and the leaves of *A*. *thaliana* expressing GFP-PTS were infiltrated with peptide/Citrine complexes or peptide/Citrine-PTS complexes.

### Cellular Uptake of Peptide/Protein Complexes

The cellular uptake of proteins was qualitatively examined by confocal laser scanning microscopy (CLSM, Carl Zeiss, Oberkochen, Germany). The intracellular delivery of the peptide/BSA-RhB complexes or the peptide/ADH-RhB complexes in YFP *A*. *thaliana* leaves was observed using the following parameters: excitation at 488 nm for the detection of YFP fluorescence, and excitation at 555 nm for the detection of RhB. To investigate the intracellular localization of Citrine and Citrine-NLS, the infiltrated wild-type *A*. *thaliana* leaves were washed twice with phosphate-buffered saline (D-PBS(-), Wako Pure Chemical Industries, Ltd., Osaka, Japan) and incubated with 800 nM 4,6-diamidino-2-phenylindole (DAPI, Sigma-Aldrich, ST. Louis, MO, USA) for 20 min under reduced pressure (approximately 0.06 MPa). The microscopy parameters were as follows: excitation at 405 nm for the detection of DAPI, and excitation at 488 nm for the detection of Citrine fluorescence. The intracellular delivery of the peptide/Citrine complexes or the peptide/Citrine-PTS complexes in transgenic *A*. *thaliana* leaves expressing GFP-PTS was observed using the following parameters: excitation at 488 nm for the detection of Citrine fluorescence and GFP fluorescence. The crosstalk of GFP and Citrine fluorescence was minimized by using the sequential scanning mode for GFP (emission range of 488 nm-510 nm) and Citrine (emission range of 570 nm-600 nm).

### Quantification of Peptide Delivery Efficiency

The infiltrated leaves of transgenic YFP *A*. *thaliana* were collected at 6 h post-infiltration with (BP100)_2_K_8_/BSA-RhB or BP100(KH)_9_/BSA-RhB complexes and then washed twice with PBS (D-PBS(-), Wako Pure Chemical Industries, Ltd., Osaka, Japan) to remove excess BSA-RhB from the surfaces of the leaves. The total crude proteins extracted from the infiltrated leaves using 1× Lysis Buffer (Promega, Madison, USA) were analyzed with 4–15% Tris glycine sodium dodecyl sulfate (SDS)-polyacrylamide gel electrophoresis (PAGE) (Bio-Rad, California, USA) at a constant 100 V. Then, the fluorescence of BSA-RhB was detected by using a luminescent image analyzer (LAS-3000, Fujifilm, Tokyo, Japan) with an excitation of 520 nm and an emission of 605 nm. After the fluorescence detection, the gel was stained with Coomassie blue G-250 (Bio-rad, California, USA) to detect the Rubisco large subunit (rbcL). The intensity of the BSA-RhB fluorescent band and the intensity of the Coomassie blue-stained rbcL band were quantified by using Image J64 (NIH, Bethesda, MD). The intensity of the BSA-RhB band was normalized to the intensity of the rbcL band. SDS-PAGE was performed again based on the normalized data. The volume of crude proteins loaded for each sample was adjusted so that the intensity of the Coomassie blue-stained rbcL band was similar for all of the samples to obtain a BSA-RhB band with normalized fluorescence intensity. Moreover, a standard curve of RhB intensity versus the known amount (micrograms) of BSA-RhB protein (positive control) was generated. The total amount of BSA-RhB protein recovered from the infiltrated leaves was calculated. The total amount of BSA-RhB protein extracted per infiltrated leaf (μg) = the amount of protein (μg) corresponding to the RhB intensity as measured per gel lane/volume of crude protein loaded per gel lane (μL) × the total volume of crude proteins extracted (μL). The percentage of extracted BSA-RhB protein per infiltrated leaf (%) = the total amount of BSA-RhB proteins extracted (μg) / the initial amount of infiltrated protein (2 μg) × 100%.

### Statistical Analysis

The quantitative data collected were expressed as mean ± standard deviation (SD) of triplicate tests. SPSS 22.0 (IBM, Armonk, NY) was used for the statistical analysis. The statistical differences were determined by Tukey’s Honestly Significant Difference (HSD) test in conjunction with the analysis of variance (ANOVA), using levels of statistical significance of *p* < 0.05.

## Supporting Information

S1 Fig SDS-PAGE of BSA-RhB prior to infiltration experiment. BSA only (lane 1), BSA-RhB (lane 2) and protein marker (lane 3). (A) The fluorescent band of BSA-RhB was detected with a luminescent image analyzer (excitation of 520 nm and emission of 605 nm). (B) The SDS-PAGE stained with Coomassie Blue G-250 in order to detect the BSA protein. BSA-RhB was detected at the molecular weight of 66 kDa. BSA was successfully labelled with RhB. The red arrows indicate the BSA-RhB.
(TIF)

S2 Fig Size distribution of the peptide/BSA-RhB complexes observed by AFM. The (BP100)_2_K_8_/BSA-RhB complexes prepared at peptide/protein molar ratio of 1 (A), 5 (B), 10 (C) and 25 (D). The BP100(KH)_9_/BSA-RhB complexes prepared at peptide/protein molar ratio of 1 (E), 5 (F), 10 (G) and 25 (H). *n* = 50.
(TIF)

S3 Fig CLSM of infiltrated leaves of *A*. *thaliana*. Transgenic YFP *A*. *thaliana* leaf after 6 hours infiltration with BSA-RhB without peptide (A), and (BP100)_2_K_8_ peptide only (B). (C) Wild-type *A*. *thaliana* leaf after 6 hours infiltration with (BP100)_2_K_8_/BSA-RhB complex prepared at molar ratio of 10. Scale bars: 20 μm.
(TIF)

S4 Fig Native PAGE of ADH-RhB prior to infiltration experiment. ADH only (lane 1 and lane 2), ADH labelled with Rhodamine B isothiocyanate (ADH-RhB) (lane 3 and lane 4), blank (lane 5), and protein marker (lane 6). (A) The fluorescent band of ADH-RhB was detected by luminescent image analyzer. (B) The native PAGE was stained with Coomassie Blue G-250 in order to detect the ADH protein. ADH tetramer was successfully labelled with RhB. The red arrows indicate the ADH-RhB tetramer.
(TIF)

S5 Fig Characterization and intracellular delivery of (BP100)_2_K_8_/ADH-RhB complexes prepared at peptide/protein molar ratio of 10. (A) Size distribution of the ADH-RhB/(BP100)_2_K_8_ complexes observed by AFM, n = 20. (B) CLSM of wild-type *A*. *thaliana* leaf after 6 hours infiltration with (BP100)_2_K_8_/ADH-RhB complex. (C) CLSM of YFP *A*. *thaliana* leaf after 6 hours infiltration with ADH-RhB without any carrier peptide. Scale bars: 20 μm.
(TIF)

S6 Fig **Size distribution of (BP100)2K8/Citrine complexes (A), (BP100)2K8/Citrine-NLS complexes (B) and (BP100)2K8/Citrine-PTS complexes (C).** The three complexes were prepared at peptide/protein molar ratio of 10 (*n* = 20).
(TIF)

S7 Fig CLSM of the leaf of transgenic *A*. *thaliana* expressing GFP-PTS after 12 hours infiltration with Citrine-PTS without peptide. Scale bars: 20 μm.
(TIF)

S1 Table Characterization data of BSA-RhB complexes of (BP100)_2_K_8_ at various peptide/protein molar ratios. (PDF)

S2 Table Characterization data of BSA-RhB complexes of BP100(KH)_9_ at various peptide/protein molar ratios. (PDF)

S3 Table Characterization data of Citrine, Citrine-NLS and Citrine-PTS. (PDF)

S4 Table Characterization data of (BP100)_2_K_8_/Citrine, (BP100)_2_K_8_/Citrine-NLS and (BP100)_2_K_8_/Citrine-PTS complexes at peptide/protein molar ratio of 10. (PDF)

S5 Table Comparison between non-covalent (electrostatic interaction) and covalent peptide-based protein delivery strategy. (PDF)
