# Supplementary figures and images for: Intracellular Delivery of Proteins via Fusion Peptides in Intact Plants
Source: PLoS One. 2016 Apr 21;11(4):e0154081. doi: 10.1371/journal.pone.0154081 (PMC4839658; doi:10.1371/journal.pone.0154081)

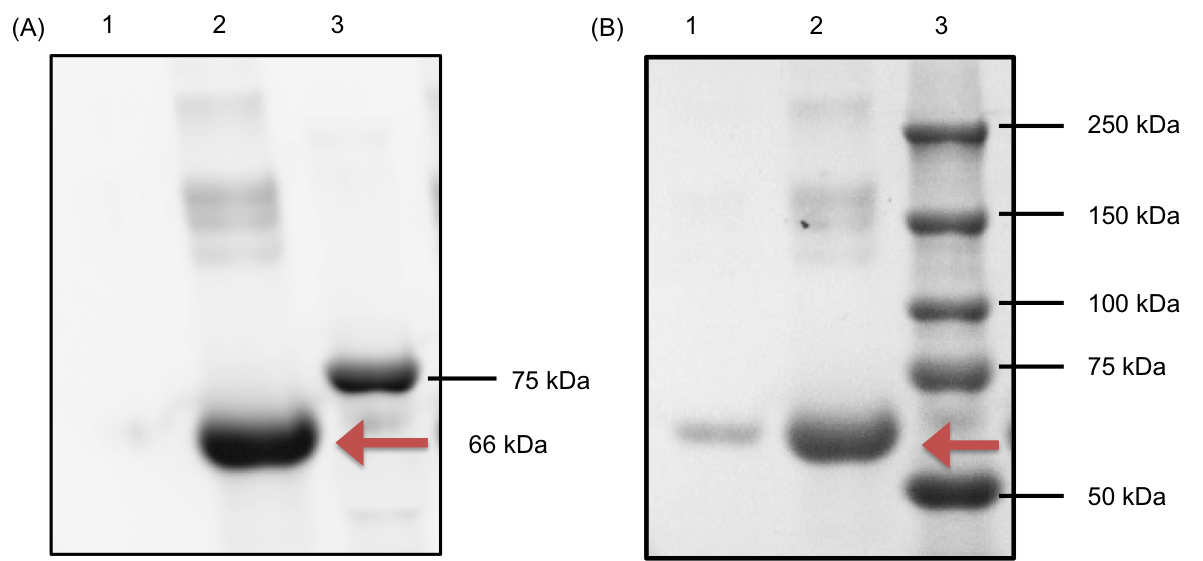

Supplement: S1 Fig — BSA only (lane 1), BSA-RhB (lane 2) and protein marker (lane 3). (A) The fluorescent band of BSA-RhB was detected with a luminescent image analyzer (excitation of 520 nm and emission of 605 nm). (B) The SDS-PAGE stained with Coomassie Blue G-250 in order to detect the BSA protein. BSA-RhB was detected at the molecular weight of 66 kDa. BSA was successfully labelled with RhB. The red arrows indicate the BSA-RhB. (TIF) [file pone.0154081.s001.tif]

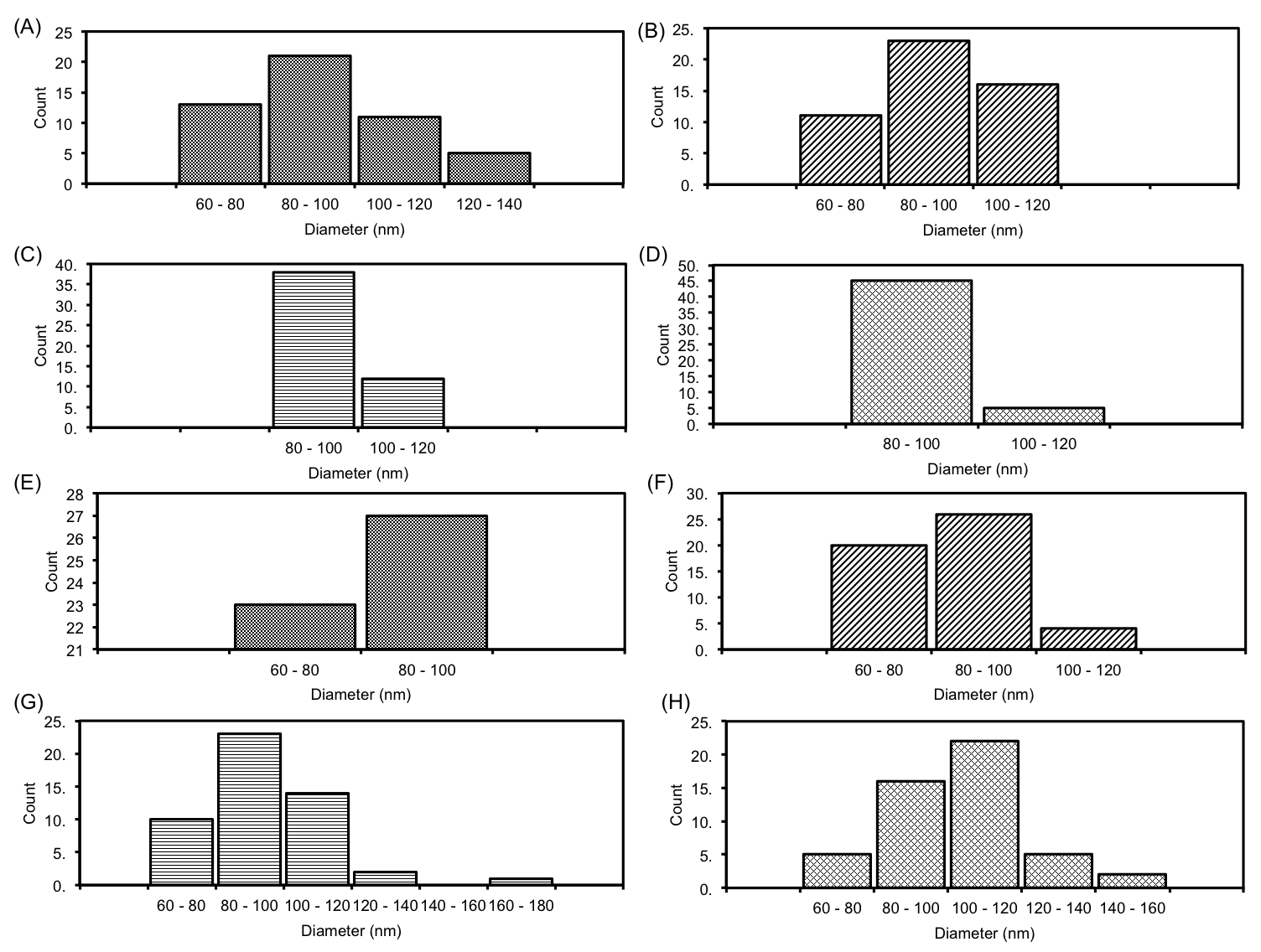

Supplement: S2 Fig — The (BP100)2K8/BSA-RhB complexes prepared at peptide/protein molar ratio of 1 (A), 5 (B), 10 (C) and 25 (D). The BP100(KH)9/BSA-RhB complexes prepared at peptide/protein molar ratio of 1 (E), 5 (F), 10 (G) and 25 (H). n = 50. (TIF) [file pone.0154081.s002.tif]

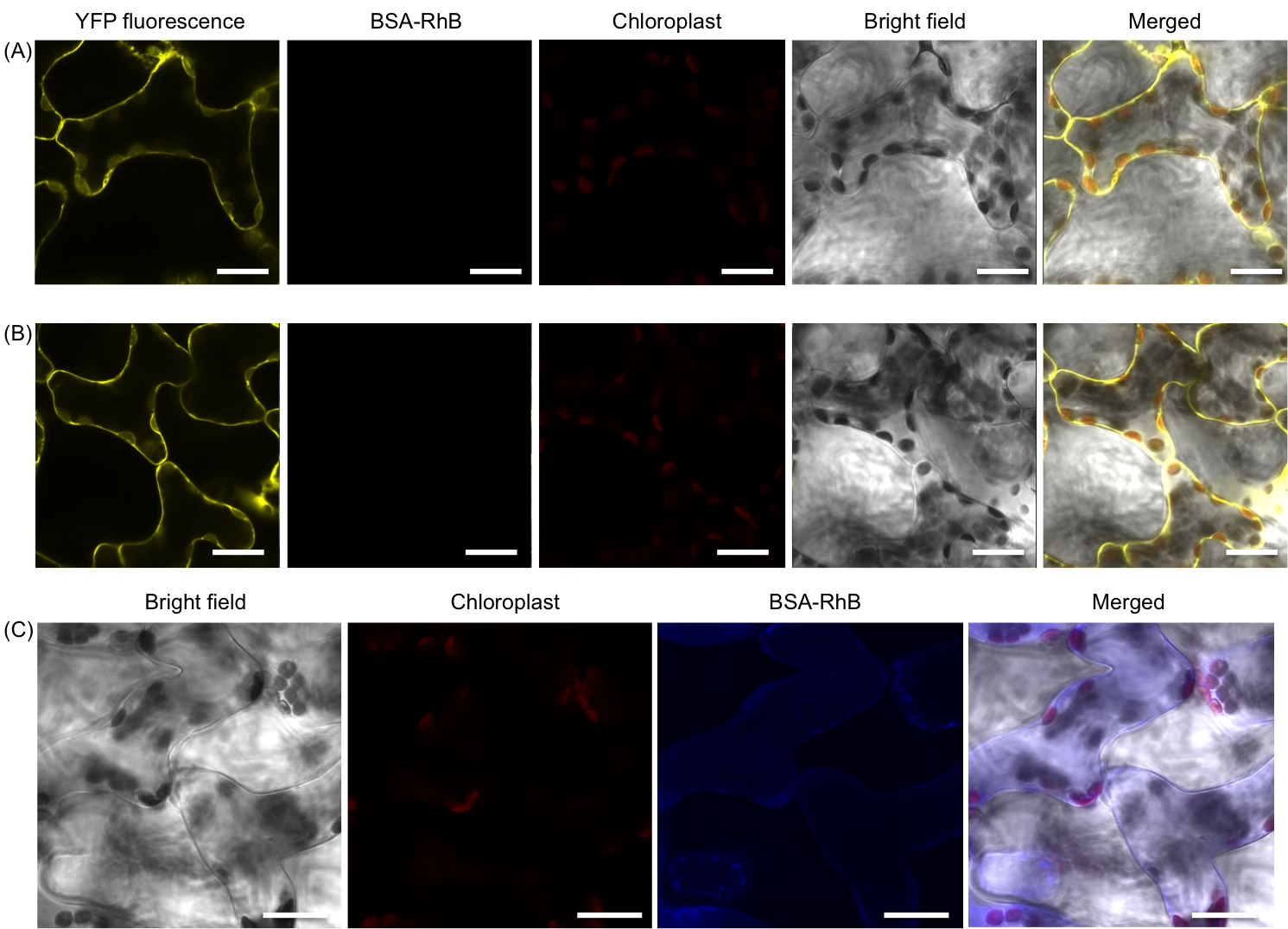

Supplement: S3 Fig — Transgenic YFP A. thaliana leaf after 6 hours infiltration with BSA-RhB without peptide (A), and (BP100)2K8 peptide only (B). (C) Wild-type A. thaliana leaf after 6 hours infiltration with (BP100)2K8/BSA-RhB complex prepared at molar ratio of 10. Scale bars: 20 μm. (TIF) [file pone.0154081.s003.tif]

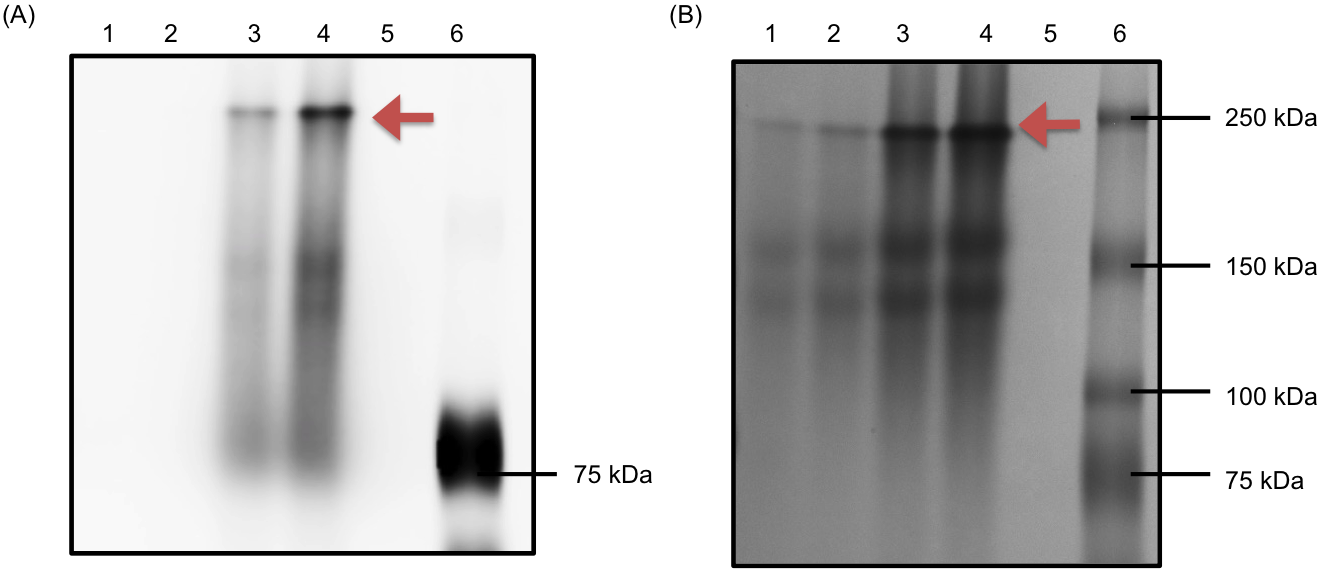

Supplement: S4 Fig — ADH only (lane 1 and lane 2), ADH labelled with Rhodamine B isothiocyanate (ADH-RhB) (lane 3 and lane 4), blank (lane 5), and protein marker (lane 6). (A) The fluorescent band of ADH-RhB was detected by luminescent image analyzer. (B) The native PAGE was stained with Coomassie Blue G-250 in order to detect the ADH protein. ADH tetramer was successfully labelled with RhB. The red arrows indicate the ADH-RhB tetramer. (TIF) [file pone.0154081.s004.tif]

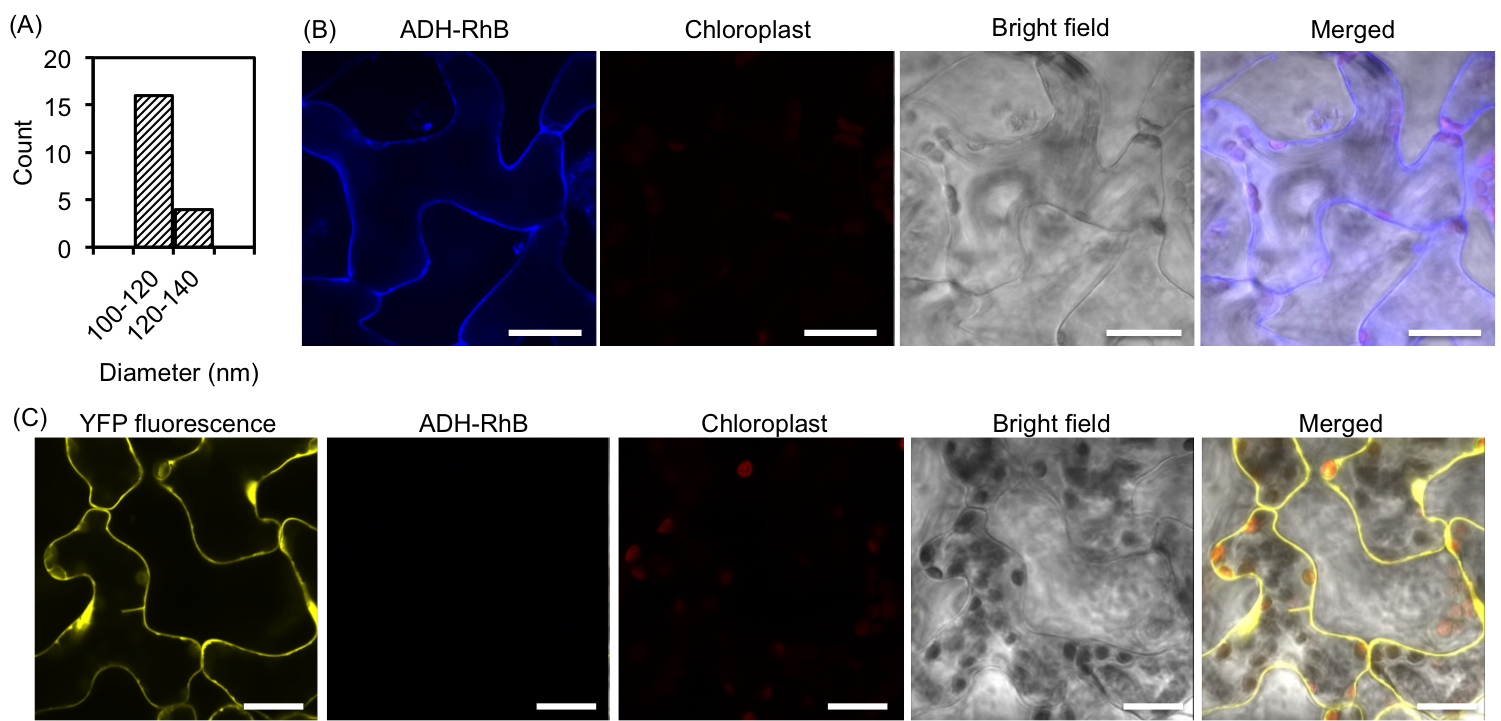

Supplement: S5 Fig — (A) Size distribution of the ADH-RhB/(BP100)2K8 complexes observed by AFM, n = 20. (B) CLSM of wild-type A. thaliana leaf after 6 hours infiltration with (BP100)2K8/ADH-RhB complex. (C) CLSM of YFP A. thaliana leaf after 6 hours infiltration with ADH-RhB without any carrier peptide. Scale bars: 20 μm. (TIF) [file pone.0154081.s005.tif]

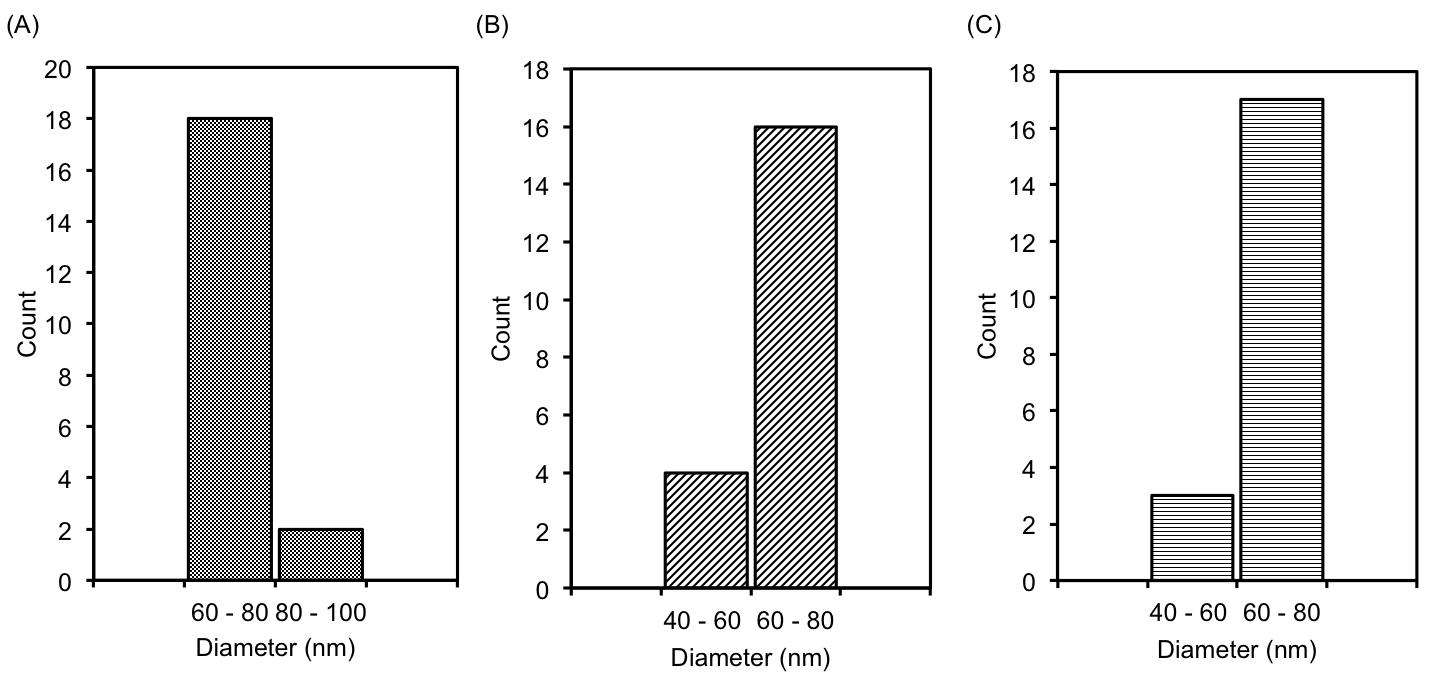

Supplement: S6 Fig — Size distribution of (BP100)2K8/Citrine complexes (A), (BP100)2K8/Citrine-NLS complexes (B) and (BP100)2K8/Citrine-PTS complexes (C). The three complexes were prepared at peptide/protein molar ratio of 10 (n = 20). (TIF) [file pone.0154081.s006.tif]

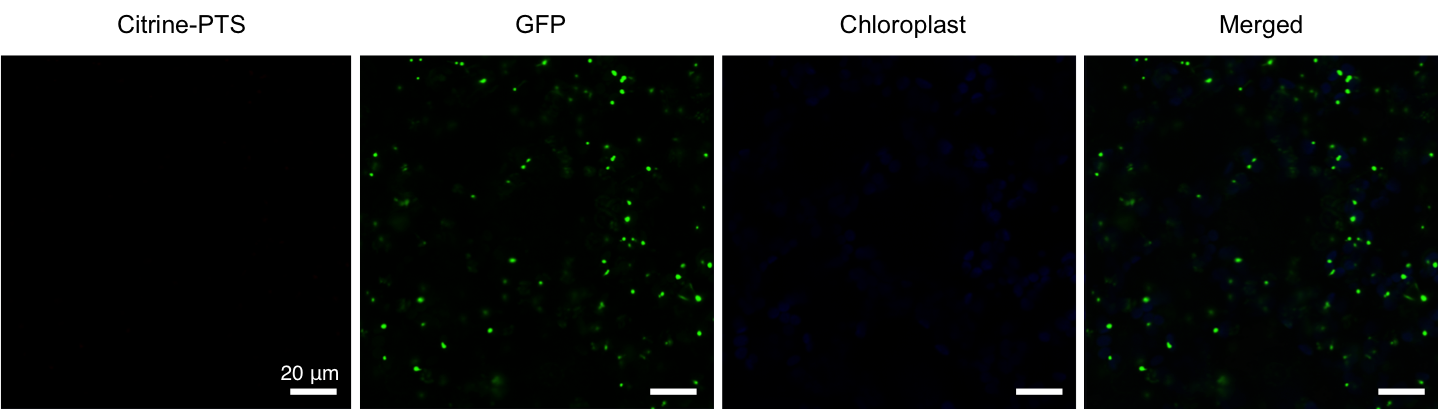

Supplement: S7 Fig — Scale bars: 20 μm. (TIF) [file pone.0154081.s007.tif]
