# Supplementary material for: Intracellular Delivery of Proteins via Fusion Peptides in Intact Plants
Source: PLoS One. 2016 Apr 21;11(4):e0154081. doi: 10.1371/journal.pone.0154081 (PMC4839658; doi:10.1371/journal.pone.0154081)
Supplement: S1 Table — (PDF) [file pone.0154081.s008.pdf]

S1 Table. Characterization data of BSA-RhB complexes of $(BP100)_2K_8$ at various peptide/protein molar ratios.

**Molar Ratios**	**Hydrodynamic diameter (nm)**	**PDI**	**Zeta potential (mV)**
1.0	341 ± 73	0.44 ± 0.05	-29.0 ± 0.2
5.0	296 ± 2	0.40 ± 0.08	18.9 ± 0.3
10.0	226 ± 1	0.15 ± 0.03	18.8 ± 0.3
25.0	173 ± 8	0.20 ± 0.01	23.4 ± 0.3
