# Supplementary material for: Intracellular Delivery of Proteins via Fusion Peptides in Intact Plants
Source: PLoS One. 2016 Apr 21;11(4):e0154081. doi: 10.1371/journal.pone.0154081 (PMC4839658; doi:10.1371/journal.pone.0154081)
Supplement: S2 Table — (PDF) [file pone.0154081.s009.pdf]

S2 Table. Characterization data of BSA-RhB complexes of BP100(KH)$_9$ at various peptide/protein molar ratios.

Molar Ratios	Hydrodynamic diameter (nm)	PDI	Zeta potential (mV)
1.0	329 ± 12	0.26 ± 0.04	-27.8 ± 0.4
5.0	452 ± 44	0.44 ± 0.02	10.4 ± 0.4
10.0	547 ± 32	0.49 ± 0.09	13.7 ± 0.2
25.0	586 ± 82	0.51 ± 0.11	15.8 ± 0.4
