# Supplementary material for: Intracellular Delivery of Proteins via Fusion Peptides in Intact Plants
Source: PLoS One. 2016 Apr 21;11(4):e0154081. doi: 10.1371/journal.pone.0154081 (PMC4839658; doi:10.1371/journal.pone.0154081)
Supplement: S3 Table — (PDF) [file pone.0154081.s010.pdf]

S3 Table. Characterization data of citrine, citrine-NLS and citine-SKL.

Proteins	Hydrodynamic diameter (nm)	PDI	Zeta potential (mV)
Citrine	188 ± 6	0.06 ± 0.02	-11.3 ± 1.5
Citrine-NLS	196 ± 7	0.03 ± 0.00	-7.37 ± 2.7
Citrine-SKL	190 ± 5	0.05 ± 0.01	-12.1 ± 1.4
