# Supplementary material for: Intracellular Delivery of Proteins via Fusion Peptides in Intact Plants
Source: PLoS One. 2016 Apr 21;11(4):e0154081. doi: 10.1371/journal.pone.0154081 (PMC4839658; doi:10.1371/journal.pone.0154081)
Supplement: S4 Table — (PDF) [file pone.0154081.s011.pdf]

S4 Table. Characterization data of $(BP100)_2K_8$/citrine, $(BP100)_2K_8$/citrine-NLS and $(BP100)_2K_8$/citrine-SKL complexes at peptide/protein molar ratio of 10.

**Complexes of $(BP100)_2K_8$**	**Hydrodynamic diameter (nm)**	**PDI**	**Zeta potential (mV)**
Citrine	263 ± 7	0.03 ± 0.03	20.1 ± 1.3
Citrine-NLS	218 ± 13	0.07 ± 0.04	23.9 ± 0.4
Citrine-SKL	253 ± 9	0.11 ± 0.04	17.9 ± 1.0
