# Supplementary material for: Intracellular Delivery of Proteins via Fusion Peptides in Intact Plants
Source: PLoS One. 2016 Apr 21;11(4):e0154081. doi: 10.1371/journal.pone.0154081 (PMC4839658; doi:10.1371/journal.pone.0154081)
Supplement: S5 Table — (PDF) [file pone.0154081.s012.pdf]

S5 Table. Comparison between non-covalent protein transduction (electrostatic interaction) and covalent protein transduction.

**Criteria**	**Non-covalent strategy (electrostatic interaction)**	**Covalent Strategy**
Preparation method	Simple and rapid (30 mins)	Time consuming and laborious (Few days)
Preservation of biological activity of protein cargoes	Yes	Potentially inhibit biological activity of protein cargoes.
Protein delivery efficiency	Not very high (12.6% in this study)	No reports on intact plants.
Versatile tool	Yes. Carrier peptide can be mixed directly with various protein cargoes of opposite charge to form peptide/protein complexes.	No. Customization is required for each type of peptide-fusion protein
